# Healthy lifestyle consultation based on traditional Chinese medicine versus routine patient education in the treatment of idiopathic sudden sensorineural hearing loss after failure of systemic therapy: study protocol for a clinical randomised trial

**DOI:** 10.1186/s13063-019-3733-5

**Published:** 2019-12-02

**Authors:** Ying-ping Fei, Yun Zheng, Dan Lai, Ping Zhong, Jing-zhe Lu, Gang Li, Peng Liu

**Affiliations:** 1 Hearing Center/Hearing and Speech Science Laboratory, Department of Otolaryngology Head and Neck Surgery, West China Hospital of Sichuan University, Chengdu, People’s Republic of China; 2 Department of Otolaryngology Head and Neck Surgery, The Affiliated Hospital of Southwest Medical University, 25 Taiping street, Jiangyang District, Luzhou, Sichuan 646000 People’s Republic of China; 3 Department of Otolaryngology Head and Neck Surgery, The First Affiliated Hospital of Guangzhou University of Chinese Medicine, No. 16 Yard, Airport Road, Guangzhou, 510405 People’s Republic of China

## Abstract

**Background:**

Idiopathic sudden sensorineural hearing loss (ISSNHL) is a major cause of deafness. Despite the advances in systemic therapy, some cases of ISSNHL are untreated, because the exact ISSNHL aetiology is unclear. Traditional Chinese medicine (TCM) has been used to treat diseases for thousands of years and is popular and widely practiced in Asia. TCM includes guidance on a healthy lifestyle. In recent decades, the relationship between lifestyle and disease has been emphasised; an unhealthy lifestyle may lead to illnesses. Thus, this study aims to compare the efficacy of lifestyle modification based on TCM with the usual consultation of ISSNHL after failure of a 2-week systemic therapy to provide a scientific basis for clinical decisions.

**Methods:**

This study is a clinical randomised trial that involves 56 patients diagnosed with ISSNHL but who have had incomplete recovery after initial management (at least 2 weeks of routine Western medical treatment). The study is performed in accordance with the sudden hearing loss clinical guideline of the American Academy of Otolaryngology–Head and Neck Surgery, published in 2012. Participants are randomly distributed into two groups: the healthy lifestyle modification group based on TCM and the control group (1:1 ratio). Patient follow-up lasts for 3 months. The primary outcome measure is the effective rate of hearing improvement, which is defined as the proportion of patients with at least 15 dB of improvement in the average thresholds of hearing loss frequency. The secondary outcome measures are improvements in word recognition score, Tinnitus Handicap Inventory and visual analogue scale for ear blockage and dizziness. Assessments are made at baseline and after lifestyle modification for 1 and 3 months.

**Discussion:**

The efficacy of healthy lifestyle modification based on a TCM programme for patients with ISSNHL with incomplete recovery after failure of initial systemic therapy is determined in this trial. Positive results will provide clinical evidence on the effects of a TCM-based healthy lifestyle, which could be recommended as salvage therapy for patients with ISSNHL.

**Trial registration:**

Chinese Clinical Trial Registry, ChiCTR-INR-17011459. Registered on 22 May 2017.

## Background

Idiopathic sudden sensorineural hearing loss (ISSNHL) is a common otologic emergency that presents mostly as an acute hearing loss with an abrupt occurrence. ISSNHL is a hearing loss of more than 30 dB that occurs in at least three consecutive frequencies within 72 h [1]. Aside from hearing impairment, ISSNHL can be associated with dizziness, tinnitus and/or ear fullness/blockage. The ISSNHL incidence is approximately 5–30/100,000/year in developed countries, such as the USA, Sweden and Japan, as revealed by national surveys [1–4]. Detailed investigations suggested that only approximately 10% of patients with ISSNHL show a specific cause [5]. Several pathophysiological mechanisms, including microcirculation, autoimmune pathology, viral infection, intracochlear membrane rupture or haematologic problems, have been proposed despite the unidentified precise cause of ISSNHL [6]. ISSNHL may not be due to a single pathological change but to a spectrum of pathologies affecting the cochlea [7].

The most common treatment option for ISSNHL is administration of corticosteroids within the first 2 weeks [4]. A total of 49–89% of patients with ISSNHL will show recovery via systemic steroid therapy, whereas therapy displays no effects on other patients [8]. Spontaneous recovery occurs in 32–65% of the cases, usually within the first 14 days [9, 10]. However, recovery amongst patients who do not show improvement after 2 weeks is low [11]. Intratympanic steroid perfusion described in the US guidelines has been recommended as salvage therapy [1]. However, its clinical evidence remains controversial, and no existing consensus suggests the efficacy of intratympanic steroid therapy for ISSNHL [1, 12, 13]. Therefore, the failure of a 2-week treatment amongst patients with ISSNHL should be further studied, and alternate therapies should be developed.

Traditional Chinese medicine (TCM) originates from ancient China and has been used in therapeutic approaches in East Asia for more than 2500 years. TCM includes well-known herbal medicine acupuncture, massage (tui na) and lifestyle modifications, such as dietary therapy and exercise (taiji and qigong). TCM originated from Huang Di Nei Jing, a famous work of ancient TCM literature, which introduced the maintenance of the Yin–Yang balance of the internal organs by following a healthy lifestyle. From the TCM perspective, all diseases originate from a broken balance. In China, Chinese patients prefer using TCM methods with complementary and alternative medicines for disease treatment. Lifestyle change guided by TCM is also acceptable amongst Chinese people with diseases.

Lifestyle change has been suggested for patients with otological diseases whose conditions are not controlled well by medicine. Dietary habits, such as a low sodium diet, can alter the inner ear’s fluid homeostasis and auditory function. The endolymph compartment maintains a low sodium concentration, and ionic balance is maintained in the surrounding perilymph and serum [14]. Evidence shows that more than 85% of patients with Meniere’s disease are helped by lifestyle changes along with either medical treatment or surgical procedures. Lifestyle changes include reducing the consumption of salt, caffeinated products, chocolate and alcohol [15]. A cross-sectional study indicates the relationship between benign paroxysmal positional vertigo and inadequate carbohydrate and fibre intake and a diet rich in polyunsaturated fatty acids. Food readjustment is suggested for patients with this condition [16]. Furthermore, a descriptive longitudinal cohort study amongst 159 adult patients with chronic primary tinnitus and sleep problems has shown that TCM-based lifestyle counselling may relieve chronic primary tinnitus. After 6–26 months of follow-up, sleep quality and tinnitus loudness improved, and the effects of tinnitus on sleep, concentration and emotional state were also alleviated [17].

Therefore, this randomised controlled clinical trial was designed to evaluate the efficacy of TCM-based lifestyle modification as a salvage therapy for patients who have not recovered 14 days after the onset of ISSNHL through the use of systemic steroid therapy.

## Methods/design

### Objective

Given the significant spontaneous recovery rate and existing standard therapy for ISSNHL by using systemic glucocorticoids, patients are enrolled in the study only if no or insufficient recovery of hearing threshold is observed after the initial 14 days of systemic therapy. In addition, patients are excluded if they are reluctant to continue receiving salvage therapy. This randomised controlled trial thus evaluates the effectiveness of healthy lifestyle treatment based on TCM therapy for patients with ISSNHL who have no or insufficient recovery of hearing threshold after the initial systemic therapy for 14 days and are reluctant to continue receiving salvage therapy.

### Study design

**Fig. 1:**
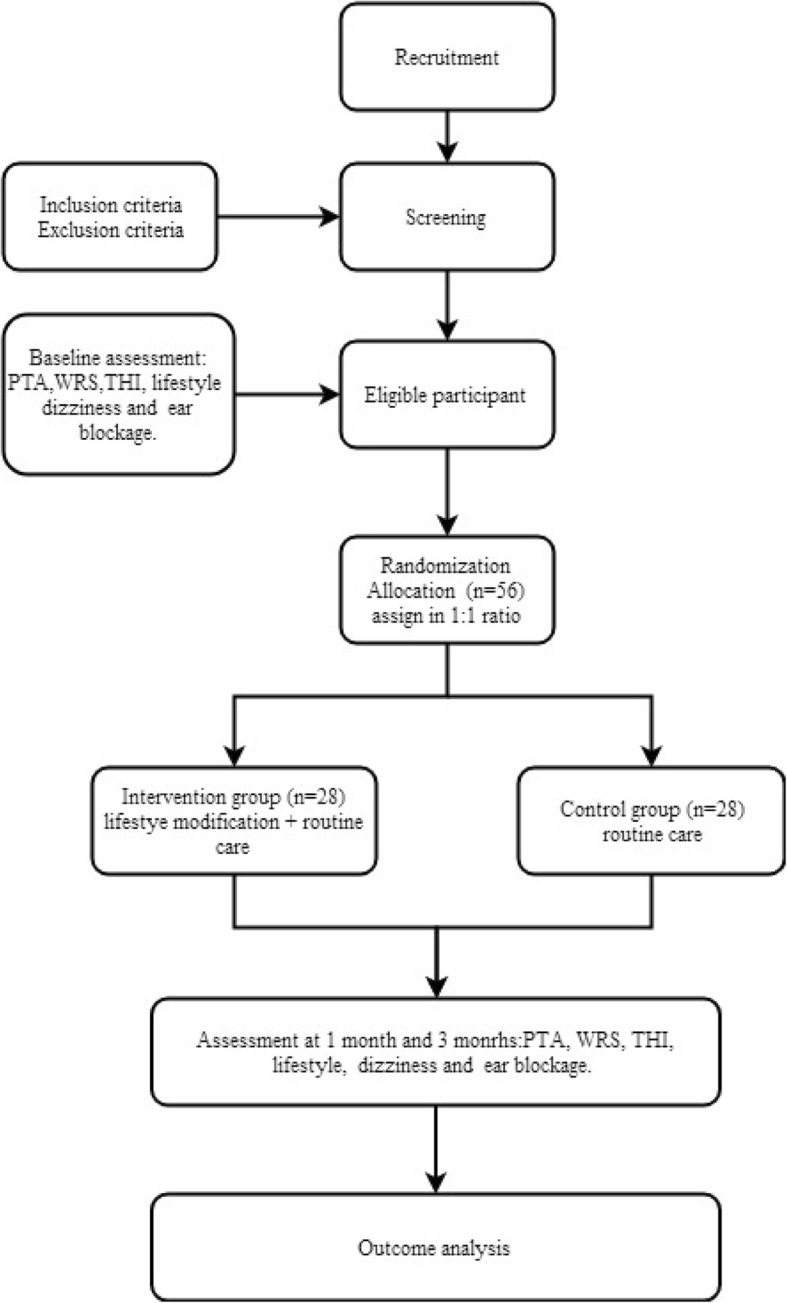
Trial flowchart. *PTA* pure tone average, *WRS* word recognition score, *THI* Tinnitus Handicap Inventory

Fifty-six patients are recruited for the trial. Participants who meet the inclusion criteria and submit written informed consent are enrolled in the trial, which lasts for 3 months. Figure 1 shows the trial procedure flowchart.

**Fig. 2:**
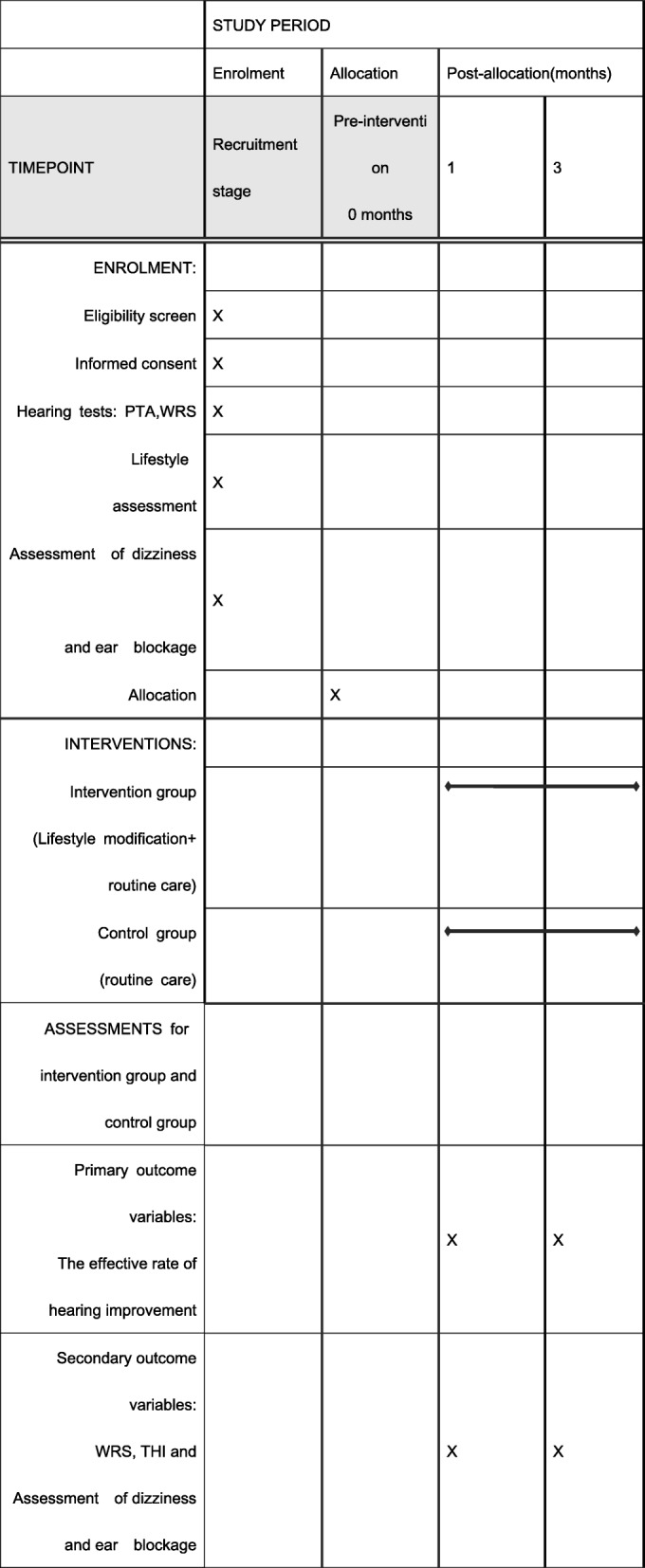
SPIRIT figure: proposed schedule for enrolment, intervention and assessment. *PTA* pure tone average, *WRS* word recognition score, *THI* Tinnitus Handicap Inventory

This trial is registered in the Chinese Clinical Trial Registry (registration number ChiCTR-INR-17011459) and has been approved by the Biomedical Branch of the Ethics Committee of the West China Hospital of Sichuan University (identifier 2016-180). The study is performed according to the Declaration of Helsinki guidelines for clinical trials. The protocol is written in line with the Standard Protocol Items: Recommendations for Interventional Trials (SPIRIT) checklist (Additional file 1), as shown in Fig. 2.

### Recruitment

Participants who are diagnosed with ISSNHL but did not respond to initial systemic treatment for at least 14 days are recruited by posters in the West China Hospital of Sichuan University.

### Participants

#### Inclusion criteria

The inclusion criteria are:

1. Signed informed consent form; participants must be willing and able to provide consent for participation
2. Diagnosis of unilateral ISSNHL, defined as onset within 72 h affecting three consecutive frequencies of unknown aetiology [1]
3. Hearing loss occuring for at least 14 days but less than or equal to 1 year
4. Insufficient recovery from ISSNHL for at least 14 days after onset and after receiving the Chinese ISSNHL guideline-recommended standard therapy
5. Reluctance to receive drugs, including steroid therapy
6. Patient age more than 18 years old but less than 60 years old
7. Hearing loss in the contralateral ear of at least 20 dB
8. Patients who stopped receiving medication for more than 3 days.

#### Exclusion criteria

The exclusion criteria are as follows:

1. Previous disease or surgery in the affected ear
2. Hearing loss from an identified aetiology, including head trauma, conductive hearing loss, Meniere’s disease or tumour
3. Inability to complete relevant assessments, such as cognitive impairment and mental disorder assessments
4. Serious comorbid conditions, such as progressive central disorder or life-threatening conditions
5. Any reason, in the investigator’s opinion, that prohibits inclusion.

The criteria for trial termination and dropout are as follows: the patient develops a severe disease unrelated to participation in the trial; the patient chooses other treatments and drugs; the patient requests termination or withdraws; the patient no longer receives the trial treatment regimen and examination.

### Randomisation

When patients want to participate in the trial and meet the requirements, a research assistant who reviews and explains the study collects the patient’s basic demographic information and previous clinical data. Eligibility of the patient is ascertained, followed by provision of the written consent form. Before randomisation, the patients must have reassessed their hearing status with an otoscopic examination, hearing threshold tests (air conduction hearing thresholds measured at 250–8000 Hz, bone conduction hearing thresholds measured at 500–4000 Hz), word recognition scores (WRSs) and ear-specific immittance measurements (including tympanometry, static immittance and acoustic reflex measures). These measures can assess the patient’s baseline hearing state and exclude conductive hearing loss. According to the classification standard of hearing loss degree published by the World Health Organization in 1997, the average values of hearing thresholds of 500, 1000, 2000 and 4000 Hz were calculated and then divided into categories of mild (26–40 dB hearing loss [HL]), moderate (41–60 dB HL), severe (61–80 dB HL) and profound (≥ 81 dB HL). Patients were stratified by gender, age and degree of hearing loss. Patients of the same gender, with age differences within 3 years and with the same degree of hearing loss were classified in the same stratification.

A statistician who is not part of the clinical intervention uses the Statistical Package for the Social Sciences (SPSS) 21.0 (IBM, Chicago, IL, USA) to generate a randomisation code. This code is embedded into serially numbered, opaque envelopes. After a participant completes the baseline measures, and when the same stratification reaches two or an even number of patients, another research assistant opens the next envelope in the series to determine the participant group allocation. Patients in the same stratification are randomised for the two treatments with a 1:1 ratio.

### Intervention

Participants in the control group receive routine care, whereas those in the intervention group receive additional lifestyle counselling based on TCM. In this system, patient care focusses on health maintenance and prevention by encouraging patients to adhere to simple health and lifestyle practices [18].

Routine care includes two aspects, as follows:

- Educating participants about the natural history of ISSNHL and the limitations of existing evidence regarding efficacy; answering patients’ questions about ISSNHL
- Counselling participants about the benefits of amplification, hearing-assistive technology and other supportive measures, especially for those whose hearing loss has lasted more than 3 months.

Lifestyle counselling consists of four sessions. The first step is the completion of the lifestyle survey of each participant and one-to-one targeted counselling based on the survey results.

- *Diet*. According to the theory of the Yellow Emperor’s Inner Canon (Huang Di Nei Jing), a classical Chinese medicine book [19], yang qi is an important reason for maintaining normal human function. In addition, a food’s energy can have a remarkable effect on health. Therefore, the diet should be dominated by staple foods, whereas ‘cold’ energy foods should be avoided. In simple terms, the central components of the dietary strategy are staple Chinese foods (with ‘neutral’ energy), such as rice and wheat. According to TCM principles, ‘cold’ energy foods include most fruits. The diet adopted in this study encourages participants to consume staple Chinese foods.
- *Sleep*. Patients sleep at night, avoid staying up late and wake up at dawn. The recommended time range for the patients to fall asleep is10 p.m. to 11 p.m., and they need to be up by 5 a.m. to 7 a.m., thereby ensuring a sleeping window between 10 p.m. and 5 a.m. Thus, reducing water intake prior to sleep is necessary to avoid waking up at night to urinate. Participants should not sleep during the day. However, a short nap of less than 30 min before 2 p.m. is advised for nonadaptive patients.
- *Mood*. A physician communicates with the participants to address their doubts and discuss the relationship between mood and ISSNHL and the importance of good mood to health. Participants’ fear, despair and anxiety should be minimal.
- *Physical activity*. All participants are encouraged to be moderately physically active by doing taiji (a traditional Chinese sport) and housework and by walking and participating in leisure activities. Patients are discouraged from engaging in deliberate strenuous physical exercise, especially before going to bed. They are also discouraged to eat too much before going to bed.

The following measures are taken to improve patient compliance and reduce the dropout rate. All participants are entitled to free assessments, including audiology tests, one-to-one consultation and lifestyle assessment. In addition, no registration fee is required for the first-level expert outpatient service from the West China Hospital. At the end of the experiment, a free online consultation service is provided for 1 year.

Weekly one-on-one consultations and periodic check-ups are provided over the phone, especially with the lifestyle modification group, to reinforce the importance of lifestyle change and answer related questions. Participants are encouraged to keep a symptom log on ear-associated and systemic symptoms. The patients need to provide daily email updates regarding their lifestyle journal, including information on sleep and wake times and daily diet (Additional file 2). Participants are required to fill in the form daily for 1 month.

### Outcome measure evaluation

The outcomes are evaluated at baseline and at 1 month and 3 months after the participant starts the intervention.

### Primary outcome measure

The primary outcome measure will be the effectiveness of hearing improvement. This will be taken as the percentage of patients with an improvement of at least 15 dB in their impaired frequency compared with the baseline. On the basis of the American Academy of Otolaryngology–Head and Neck Surgery 2012 practice guideline on ISSNHL, an improvement within 10 dB HL of initial HL or within 10 dB HL of the unaffected ear’s hearing threshold is defined as complete recovery. An improvement of more than 30 dB HL in pure tone average (PTA) from pretreatment hearing levels is defined as significant recovery; an improvement of 15–30 dB HL is defined as effective recovery. An improvement of less than 15 dB HL in PTA is defined as no recovery [1, 20].

### Secondary outcome measures

Based on the visual analogue scale, the secondary outcomes include improvement of adherence to TCM lifestyle and evaluation of changes in WRS [21], Tinnitus Handicap Inventory (THI) [22] for patients with tinnitus and change in common symptoms, such as dizziness and ear blockage. These outcomes are measured at the first visit and during protocol visits.

### Blinding

The audiologist, research assistants and statisticians involved in the study are blinded to the allocations. Given the nature of the counselling, blinding amongst consultants and patients is impossible. Thus, consultants and other researchers do not communicate amongst one another about the patient group during the trial. Patients also keep their treatment methods confidential. At the completion of the trial, patients in the control group are offered access to the lifestyle modification intervention.

### Sample size

To the best of our knowledge, no randomised pilot study has been conducted to assess the effectiveness of lifestyle changes on ISSNHL. Therefore, we are not able to calculate the sample size based on previous studies. On the basis of our retrospective analysis (unpublished) and clinical experience, the efficiency ratio of the intervention group is conservatively estimated to be 50%, and the natural recovery rate over 2 weeks is 10% [9]. Using a formula [23] to calculate the sample size of optimal treatment in the clinical trial and considering α = 0.05, β = 0.1, by the table of normal distribution quantifiers *U*_α(0.05)_ = 1.65, *U*_β(0.1)_ = 1.28, we require a patient sample size of 23 per group. We allow for a 20% loss to follow-up (approximately 10 cases), with a total sample size of 56 patients (28 per group) in the study.

$$ {\displaystyle \begin{array}{l}n={\left( U\alpha + U\beta \right)}^22P\left(1-P\right)/\left({P}_1-{P}_0\right)2,\\ {}P=\left({\mathrm{P}}_1+{\mathrm{P}}_0\right)/2\times 100\%,\end{array}} $$

*P*_0_: original efficacy, *P*_1_: expected efficacy.

### Safety

Any adverse events or discomfort throughout the course of the trial will be recorded by patients and data collectors. Participants may withdraw from the study for any reason at any time. The researchers will record the reasons on case report forms.

### Statistical analysis

Data are analysed using SPSS V.21.0 (Chicago, IL, USA), with the significance level set at 0.05 (two-tailed) by statisticians who are independent of the research team. Patient baseline characteristics are summarised by treatment arm by employing appropriate summary statistics to assess baseline comparability only. Data analysis is conducted with the intention-to-treat (ITT) principle and a per-protocol (PP) analysis. To ensure the comparability of baseline conditions between the two groups and allow the presence of noncompliant patients, the ITT population consists of all randomised participants. In addition, at least one follow-up will be recorded after the intervention. According to the patients’ actual lifestyle adjustment after random grouping, the PP analysis studies the patients who completed the trial and did not violate the protocol. This process is an explanatory, supplementary analysis, contributing to study objectivity. We calculate the effective rates at 1 and 3 months for the primary outcome and compare the intervention and control groups by using the χ^2^ test. For secondary outcomes, continuous variables, including THI, visual analogue scale and WRS, are compared between the two groups at all follow-up time points by using a *t* test or the Wilcoxon signed-rank test as appropriate. Categorical variables, such as different degrees of hearing loss, are compared using the χ^2^ test or Fisher’s exact test. For dropout analysis, we use multiple imputations for ITT analysis. A sensitivity analysis is performed to assess the effect of missing data assumptions.

### Data management

Data accuracy is ensured by completing the paper copies of the case report form. Two independent researchers blinded to the group allocation input the data on an Excel spreadsheet, and the data are checked twice. Data are validated using original case report forms when any discrepancy is discovered. Paper files and electronic documents are stored separately in a locked filing cabinet and on a protected computer. Only the principal researchers are allowed access to the data. Researchers are unable to modify the data. They shall keep the information strictly confidential and shall not disclose it under any circumstances. The researchers shall sign a confidentiality agreement.

## Discussion

ISSNHL, for which the aetiology remains unknown, is an acute disorder that occurs throughout life. Although 49–89% of patients achieve normal hearing through existing therapy with oral or intravenous steroids and with 32—65% spontaneous recovery rates, treatment amongst patients who have incomplete recovery from ISSNHL after failure of initial management remains a problem [8–10]. Some patients are reluctant to receive recommended glucocorticoid treatment because of concerns regarding side effects, contraindications and drug-to-drug interactions. The salvage therapy recommended by the 2012 ISSNHL guidelines is steroid perfusion, which results in hearing improvement ranging from 53 to 90% in the treatment group [24, 25]. The dose and concentration of steroids vary similarly to the criteria used to define hearing improvement.

Previous studies indicated that patients who do not show any improvement within the first 14 days are unlikely to show remarkable recovery afterwards [9, 10], and thus they usually lose hope and discontinue therapy. Therefore, an acceptable and simple therapy is required to improve the effects of refractory ISSNHL for these patients.

TCM has been used for thousands of years and is widely accepted for treating diseases in China. Lifestyle, as part of TCM, has been integrated into the Chinese culture. Tradition and sustained interest in the benefits of a TCM-recommended lifestyle have remained, especially for patients whose conditions have not been effectively treated by Western medicine. We designed this trial to verify that a TCM-based lifestyle can provide help concerning hearing loss and concomitant symptoms amongst patients with ISSNHL. If successful, this intervention may help patients with refractory ISSNHL in China.

The study is designed to explore the efficacy of a TCM-based lifestyle change for patients with ISSNHL who have no or insufficient recovery after initial systematic Western medicine treatment. Although intratympanic steroid perfusion for this kind of ISSNHL has been used in Western treatment, the evidence of its efficacy remains unclear. In this study, the effective rate of hearing improvement is used as the primary outcome measure and is the most common parameter for ISSNHL. Secondary outcome measures include WRS, THI and accompanying symptoms, including dizziness and fullness of the ear. In addition to hearing loss, many people with ISSNHL complain of tinnitus and dizziness. The exact mechanisms that explain the effects of a TCM-based lifestyle change on ISSNHL require further detailed research and discussion in the future.

### Study limitations

One of the major drawbacks of this study is that the currently widely adopted lifestyle questionnaire is not used. Lifestyle includes sleep, diet, mood and exercise. Thus, some factors that play a key role in improving ISSNHL remain unknown. Therefore, well-designed and randomised controlled trials that compare different lifestyle factors with one another are necessary in the future.

### Trial status

The trial is currently recruiting patients. We have completed patient recruitment in October 2019.

## Supplementary information

**Additional file 1.** SPIRIT checklist: recommended items to address in a clinical trial protocol and related documents.

**Additional file 2.** Lifestyle diary. Participant completes the table to record a journal of sleep time and daily diet.

## Data Availability

The datasets used and/or analysed during the current study are available from the corresponding author on reasonable request.

## Abbreviations

ISSNHL: Idiopathic sudden sensorineural hearing loss
PTA: Pure tone average
TCM: Traditional Chinese medicine
THI: Tinnitus Handicap Inventory
WRS: Word recognition score
