# Supplementary material for: Healthy lifestyle consultation based on traditional Chinese medicine versus routine patient education in the treatment of idiopathic sudden sensorineural hearing loss after failure of systemic therapy: study protocol for a clinical randomised trial
Source: Trials. 2019 Dec 2;20:666. doi: 10.1186/s13063-019-3733-5 (PMC6889698; doi:10.1186/s13063-019-3733-5)
Supplement: Supplementary file 2 — Additional file 2. Lifestyle diary. Participant completes the table to record a journal of sleep time and daily diet. [file 13063_2019_3733_MOESM2_ESM.docx]

**Lifestyle Diary**

**Name: ID: Gender: Birth date: Fill-in-Date:**

**Please fill the following form to record your lifestyle from *8pm yesterday* to *8pm today*.**

**Please fill in the blanks “___”or click the answer with “**√**”.**

1. **Foods & Drinks:**

**(1) Do you have good appetite while eating OR do you enjoy eating?**

**(2) Compared with yesterday, is your appetite better, same, or poorer today? Better Same □Poorer**

| **Foods & Drinks** | **Time you start & end eating** | **What did you eat & drink? Please list as detailed (preferably, you could include quantity of your foods, how you cook your foods, etc. for three meals) as you could, like the examples given below.** | **% of the main foods*** | **% of animal foods** among foods that are not main foods during each of your 3 meals.** |
| --- | --- | --- | --- | --- |
| **Breakfast** |  |  |  |  |
|  |  |  |  |  |
| **Lunch** |  |  |  |  |
|  |  |  |  |  |
| **Dinner** |  |  |  | **.** |

***** **Main foods: Any foods made of purely rice or flour.**

**** Animal foods: Any foods that are or might contain animal protein, e.g., you cannot have bread with butter and eggs, soup cooked with seafoods, vegetables cooked with fish.**

|  | **Foods & Drinks that you have had at times other than the above 3 meals.**  **(including nutrition supplements)** |
| --- | --- |
| **1** |  |
| **2** |  |
| **3** |  |
| **4** |  |
| **5** |  |
| **6** |  |
| **7** |  |
| **8** |  |

1. **Stool and urine**

|  | **& urine** |  | **Please fill in the blanks “___”or click the answer with “√”.** |
| --- | --- | --- | --- |
| **Stool** | **Time you start & end stool each time?** | **How many times a day?** | **Normal stool is yellow or golden yellow and the shape is like banana.** |
|  |  |  | **Color: Yellow □Brown □Dark brown □Black □Green □Grey white □Others:** |
|  |  |  | **Shape: □Soft and like banana** [**Thin**](about:blank) [**sloppy**](about:blank) [**stool**](about:blank) **□Like water □Contain undigested food □Hard and like stool of sheep …** |
|  |  |  | **Bloody:** **□Yes No** |
|  |  |  | **Mucopurulent: □Yes No** |
|  |  |  | **Painfully hard: Yes □No** |
|  |  |  | **If you find the above is hard to fill in, please describe your stool here with your own words:** |
| **Urination** |  | **How many times per night during sleep?** | **Normal urine is light yellow and clear.** |
|  |  |  | **Color: X Light yellow □yellow □Brown □Red □White** |
|  |  |  | **Transparency:□Clear X Slight Turbidity** |
|  |  |  | **Urgent: X □No** |
|  |  |  | **Painful: X No** |
|  |  |  | **If you find the above is hard to fill in, please describe your urination here with your own words:** |

**Sleep –**

**When did you get to bed last night? Went to bed at**

**When did you get up this morning? am**

**Did you fall asleep easily last night Yes/No**

**How many times did you wake up last night?**

**Did you fall asleep again easily?**

**Did you sleep during daytime?**  **(When and how long did you sleep? Please describe:**

**Mood**

**Did you have a good mood? Was good most of the day**

**Do you feel energetic? My energy was good**

1. **Physical exercise**

**Did you have set aside special time for physical exercise? (When and how long did you exercise? What kind of exercise did you do? Please describe:**

**Did you sweat a lot after exercise? □Yes X No**

**Symptom Diary**

**Instructions:**

**Please arrange your symptoms according to how much you care about them in the following table. Give number 1 to the symptom that you care about the most, number 2 you care about the second most, etc. e.g., If you tinnitus is the symptom you care about the most, give it a number 1, if hearing loss is the second most symptom you care about, give it a number 2. Please list all your symptoms from your head to your feet that makes you uncomfortable, not just discomforts in the ears. Finally, please check the cell with a "√" to indicate how each of your symptoms changes every day.**

| **No. of your Symptoms** | **Symptom(s)** | **Better than yesterday** | **Same as**  **yesterday** | **Worse than**  **yesterday** | **Other comments** |
| --- | --- | --- | --- | --- | --- |
| **1** | **Headaches/**  **Neck and back tension** |  |  |  |  |
| **2** | **Hot flashes** |  |  |  |  |
| **3** | **Uncomfortable stomach/GI tract** |  |  |  |  |
| **4** | **Heart concerns or irregularities** |  |  |  |  |
| **5** | **Low energy** |  |  |  |  |
| **6** | **Low brain function – lethargic like** |  |  |  |  |
| **7** | **Low mood** |  |  |  |  |

**Healthy lifestyle is the key to cure of your discomfort.**

**Keep up with healthy lifestyle one day, you will be one day more closer to final complete health!**

**We are looking forward to hearing from your good news every day!**
